# Peritrichs (Ciliophora, Peritrichia) in the Danube: Keystone Organisms in the Formation of Diverse Protist Biofilms

**DOI:** 10.1111/1758-2229.70215

**Published:** 2025-10-15

**Authors:** Álmos Becz, Júlia Katalin Török

**Affiliations:** ^1^ Doctoral School of Biology, Institute of Biology ELTE Eötvös Loránd University Budapest Hungary; ^2^ Department of Systematic Zoology and Ecology, Institute of Biology ELTE Eötvös Loránd University Budapest Hungary

**Keywords:** biofilm, colonisation, epibionts, functional groups, large rivers, peritrichs, seasonal pattern

## Abstract

Peritrichs are widely studied ciliates; however, their eukaryotic epibionts have not yet been examined in detail. Our study investigates the colonisation patterns and seasonal dynamics of peritrich ciliates and their epibionts in lotic environments over 14 sampling periods. In the early stages of colonisation, settlement was likely characterised by random establishment, consistent with the lottery model of Chesson and Warner. In later stages, the autogenic, ecosystem‐engineering role of peritrich species facilitated the settlement of additional organisms on the substrate. During warmer months, the structurally complex surface of peritrichs hosted a greater abundance of epibionts—particularly choanoflagellates. Colonisation of the peritrich stalks by attached filter feeders and other functional groups increased the filtration‐capable surface area, thereby enhancing biofilm function. Additionally, the presence of predatory ciliates such as *Trachelius ovum* can alter biofilm structure by consuming colonial peritrichs. These findings highlight the crucial role of peritrichs in biofilm dynamics and their contribution to community complexity in lotic ecosystems.

## Graphical abstract

Colonial peritrichs act as physical ecosystem engineers in middle Danube biofilms, generating three‐dimensional structures during 1–28 days of colonisation. Their surfaces host filter feeder epibionts (choanoflagellates, other peritrichs, rotifers), expanding filtration capacity and promoting functional group establishment including swimming and attached predators, especially in warmer environments. By Days 11–28, functional redundancy emerges, enhancing community resilience.

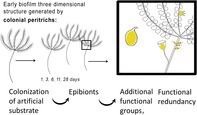

## 1 Introduction

Large rivers play a pivotal role in the global carbon cycle through the heterotrophic organisms they host (Battin et al. 2023; Talluto et al. 2024). Heterotrophic protists occupy a fundamental position in the food webs of natural aquatic environments and contribute significantly to the mineralization of organic matter (Fenchel 2008; Psenner et al. 2008; Okuda et al. 2014). However, the precise role of various protist groups within food webs remains poorly understood, even in freshwater habitats that have been extensively studied from this perspective (Sommer et al. 2012).

Biofilms are particularly worthy of investigation due to their microbial activity, which significantly exceeds that of the surrounding water column (Costerton et al. 1995). In running waters, the microbial biofilm in the riparian zone plays a crucial role in the ecosystem's carbon cycle, facilitated by vegetation that provides a large surface area (Pusch et al. 1998). Among the microorganisms in biofilms, most research has focused on diatoms (Zhao et al. 2023). However, the precise role of heterotrophic microeukaryotes within biofilms remains insufficiently explored, and further research is needed to investigate interspecific trophic relationships (Weitere et al. 2018; Reiss 2021). Of the microeukaryotes, ciliates represent one of the most extensively studied groups (Clamp and Lynn 2017).

The colonisation of heterotrophic biofilms has been studied in both freshwater and marine environments. Several studies conducted in these environments have observed the MacArthur‐Wilson species accumulation curve—a pattern commonly associated with colonisation processes at larger spatial scales—during the colonisation process (Cairns Jr et al. 1969; Azovsky 1988; Zhang et al. 2012). However, significant knowledge gaps remain regarding the colonisation process. In particular, it is not yet well understood which factors determine the order of settlement of representatives from different guilds during colonisation. Furthermore, it is important to highlight that there is limited research on the colonisation of biofilms in lotic environments (Kusuoka and Watanabe 1987; Primc and Habdija 1987; Harmsworth and Sleigh 1993; Risse‐Buhl and Küsel 2009). However, only a small proportion of the experimental studies conducted in running water environments focus on riverine contexts (Nosek and Bereczky 1983; Bereczky 1990; Früh et al. 2011; Ackermann et al. 2011).

Among the ciliate protists in biofilms, many species adopt a filter‐feeding lifestyle, and these species can have a significant impact on plankton through their filtration activity (Weitere et al. 2003). Peritrichs (Oligohymenophorea: Peritrichia) are often the most abundant ciliate protists in biofilms (Ackermann et al. 2011; Kathol et al. 2011, 2009; Martin‐Cereceda et al. 2001; Vlaičević et al. 2017). However, peritrichs contribute to biofilms not only through their abundance but also by enhancing their complexity through taxonomic diversity. More than a thousand species of peritrichs are believed to exist, with approximately 800 species currently documented within the Sessilida order (Lynn 2008; Sun et al. 2011; Wang, Jiang, et al. 2022; Wang, Feng, et al. 2022). Information is available on the seasonal occurrence of more common Peritrichia species (Foissner et al. 1992). However, there are very few case studies that report the Peritrichia patterns of a specific habitat over the course of an entire year (Mieczan 2010; Safi et al. 2014; Vlaičević et al. 2017, 2021; Abdullah Al et al. 2018). Even fewer studies present results from sampling conducted in temperate riverine environments (Kathol et al. 2011).

In this study, we monitored for the first time the colonisation of biofilms by peritrich ciliates and other microeukaryotes on artificial substrates in the Soroksár branch of the Danube River over 14 study periods ranging from 1 to days. Previous studies have focused on the planktonic heterotrophic protists of the middle section of the Danube (Nosek and Bereczky 1994; Kiss et al. 2009); however, further investigations in this area have not been conducted. The scientific literature contains limited information on the biofilms of the Danube (Ertl 1970; Krno et al. 1999), and data on biofilm colonisation patterns in the Danube are restricted to a few months (Bereczky 1990).

Foissner and colleagues have compiled extensive literature on the various protists and rotifers capable of colonising different peritrich species, supplementing this information with their own observations (Foissner et al. 1992). Matthes' (1982) study also reported peritrichs and suctorians colonising other peritrichs. Preliminary observations of microeukaryotes colonising sessile peritrichs have already been made at the study site (Becz and Török 2023). However, no studies have yet been published in the literature regarding the seasonal patterns of microeukaryote colonisation on the surfaces of sessile peritrichs.

The aim of our research was to produce a novel and comprehensive study on the colonisation patterns and seasonal dynamics of biofilms in a riverine environment. We monitored colonisation patterns from the early stages of biofilm development through to the climax state. The latter we define as a peritrich‐based biofilm characterised by a well‐established peritrich community and without an excessive abundance of bacteria, algae, or microfauna. The seasonal dynamics of the biofilm were investigated over a period exceeding 1 year.

The most comparable environment to our study site, located in the Rhine, was examined by Kathol et al. (2011), and we thus expect similar patterns to emerge. An additional objective was to examine whether the species turnover rates observed during early colonisation qualitatively align with the expectations of the lottery model, acknowledging that no direct quantitative comparison with the model was performed. According to the lottery model by Chesson and Warner (1981), the maintenance of diversity in a given area is significantly influenced by the random occupation and settlement of different species' dispersal structures. We hypothesize that the colonisation by colonial peritrichs, which are considered long‐lived in their environment and possess dispersal structures (e.g., swarmers), is well approximated by the lottery model.

The term ‘ecosystem engineer’ was coined by Jones et al. (1994) to describe organisms that alter resource availability for other species by transforming biotic or abiotic materials and thereby creating and modifying habitats. Jones et al. (1997), later Jones et al. (2010) expanded the concept by demonstrating that the physical modifications performed by an ecosystem engineer can generate feedbacks that affect its own ecological niche, population dynamics, and evolutionary trajectory. Over time, colonial peritrichs form colonies with high structural complexity within the biofilm, which can be easily colonised by other protists. In the later stages of colonisation, we hypothesize that physical ecosystem engineering (Jones et al. 1994) may represent a characteristic feature of peritrich biofilms.

The growing season, which is typically characterised by higher temperatures, has better food availability (bacteria, algae) compared to the colder months of the year (Besemer et al. 2009; Kathol et al. 2011). Therefore, we hypothesize that during this period, other microeukaryotes that colonise peritrichs in significant abundances contribute new functional groups within the biofilm.

## 2 Material and Methods

### 2.1 Study Site

The collection took place in the Soroksár Danube, situated south of Budapest, near Szigetszentmiklós (N47.339816°, E19.050299°). This location was identical to the one used in a previous study (Becz and Török 2023). The Soroksár Danube is a slow‐flowing sidearm of the Danube, where the water flow is between 0.2 and 0.4 km/h, and the water body is characterised by abundant macrophyte vegetation (Tóth 2014; Czira, Czira et al. 2023). The samplers were placed under a willow tree in a shaded area, as light exposure may influence the development of protozoan communities (Bengtsson et al. 2018; Naik et al. 2022).

### 2.2 Sampling and Enumeration

Sampling was performed between December 2020 and November 2023. We conducted a series of short, 28‐day colonisation experiments in succession. During each colonisation period, samples were collected on days 1, 3, 6, 11 and 28. A total of 14 consecutive colonisation periods were examined, covering a span of more than 1 year. In total, 70 sampling events were conducted, each with three biological replicates, resulting in a total of 218 samples analysed. During the investigation, instances of data collection with missing information were documented in the table included in the Supporting Information, indicating the extent of the data gaps (Table S1).

The sampler was composed of a submerged solid surface to which glass slides were attached. To implement the parallel probes, three separate samplers equipped with buoys were used as biological replicates during each sampling event, ensuring sample independence.

On each colonisation day, one glass slide was randomly collected from each of the three buoys. Each collected slide was transported to the laboratory in a separate 50 mL Falcon tube. Four quadrats on each collected slide were examined, located beneath the 24 × 40 mm cover slip, randomly distributed across the upper (A quadrat), middle (B and C quadrats) and lower regions (D quadrat) of the slide (Figure S1). The area of each quadrat was 41.7 mm^2^. The total area of the quadrats was documented through photographs taken at 100× magnification using a Zeiss Primostar microscope equipped with a Panasonic GH5 camera. Photographic documentation of live samples was carried out up to 13 h after collection. The rapid examination was necessary to minimise changes in community structure (Zhang et al. 2012). Photo and video documentation of live samples is an important tool for better understanding trophic interactions (Weitere et al. 2018).

During data collection, abundance data were obtained by averaging the four quadrats for each parallel probe. No AI‐based or other software was used to extract abundance data from the photo and video documentation; instead, cell counts were performed manually by the researcher (Á.B.) for each individual image. In our study, we also counted the remains of dead peritrich colonies (those without zooids) in order to analyse the effects of predators.

The identification of ciliates was carried out following Stiller (1971) and Foissner and Berger (1996). The taxonomic classification of the identified ciliates was based on Lynn (2008), while other protists were classified according to Lee et al. (2000).

During the study, colonial peritrichs were identified to the genus and species levels. In addition, all protists that either colonised the surface of sessile peritrichs or were observed swimming in the water column and capable of feeding on peritrichs were identified to genus and species levels whenever possible. These latter protists belonged to the following groups: Ciliophora, Choanoflagellatea, Euglenida, and Centrohelida.

**FIGURE 1:**
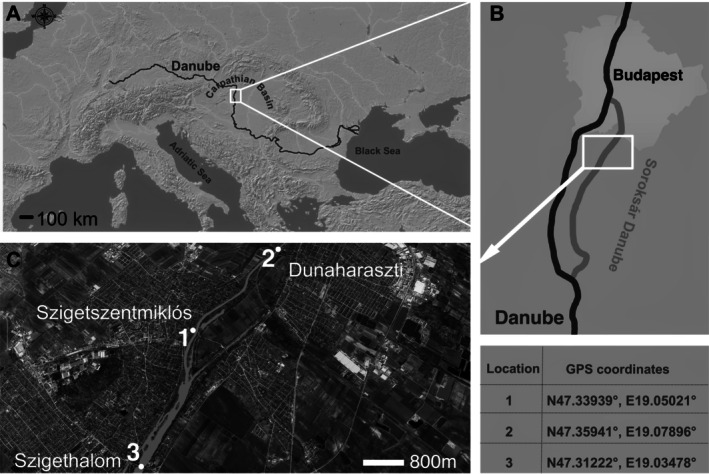
Map illustrating the study area, situated in the Carpathian Basin, adjacent to the Soroksár section of the Danube River. The Danube River in Europe (A). The Soroksár branch of the Danube River (B). Satellite image of the sampling site (C). Sampling and monitoring sites: (1) biofilm collection; (2 and 3) governmental environmental monitoring.

The environmental variables (Water T (°C), pH, electric conductivity (EC, μS/cm), dissolved oxygen (mg/L), oxygen saturation (%), chemical oxygen demand (COD, mg/L), mineral N (mg/l), phosphate‐P (μg/L)) were obtained from the Middle Danube Valley Water Directorate, a Hungarian governmental authority responsible for water quality monitoring. These variables were recorded from locations 3 km upstream (Dunaharaszti) and 3 km downstream (Szigethalom) from the sampling point in Szigetszentmiklós (Figure 1, Table S2).

Environmental measurements carried out by the authorities were conducted at two‐week intervals. The environmental variables determined according to specific Hungarian Standard (MSZ) were as follows: Water temperature: MSZ 448‐2:1967; pH: potentiometry, MSZ 1484‐22:2009; EC: conductometry, MSZ EN 27888:1998; Dissolved oxygen: iodometry, MSZ ISO 5813:1992; Oxygen saturation: MSZ EN ISO 5814:2013; Mineral nitrogen: spectrophotometry, MSZ 448‐27:1985; Phosphate‐P: spectrophotometry, MSZ 12750‐17:1974. COD was measured spectrophotometrically according to ISO 15705:2002.

For each colonisation day, the three temporally nearest consecutive measurements from both upstream and downstream sites were averaged, providing a single representative value of each environmental variable for each biological sample.

### 2.3 Assignment of Protists Into Ecological Functional Groups

During the investigation, the identified taxa were classified into functional groups based on their feeding strategies and positions within the biofilm. The functional group classification and its literature sources are summarised in Table S3. The position of the various taxa—whether attached to the glass substrate or to the surface of a peritrich—was determined during our own microscopic observations. For our analyses, we applied the groups resulting from the combination of these two functional classifications. The use of such combined functional groups, established on a similar principle, has already proven to be an effective method in phycology (Tapolczai et al. 2016; Wang et al. 2018). Members of the ‘AN’ group were collectively referred to as epibionts.

### 2.4 Statistical Analyses

The statistical analyses were performed using RStudio (Posit Team 2024). The classification of glass‐attached colonial peritrichia samples was performed using cluster analysis with the ‘pvclust’ package, applying the UPGMA method and Euclidean distance (seed: 123, nboot = 999) (Suzuki et al. 2015).

The relationships between protist communities across the various sampling months and the association between environmental variables and protist communities was investigated by dbRDA ordination with the ‘vegan’ and ‘permute’ package (distance: Bray‐Curtis, seed: 999, nboot = 9999, steps = 20,000) (Simpson 2022; Oksanen et al. 2024). The distinction between a priori groups (designated according to the results of cluster analysis) was examined using the non‐parametric statistical test ANalysis Of SIMilarities (ANOSIM) (perm = 9999). Taxon richness, Shannon diversity, evenness and abundance were calculated for each colonisation day using the ‘vegan’ package.

Additionally, the relationship between the cube root transformed cumulative proportion of epibionts and temperature was investigated using linear regression with the ‘vegan’ package.

To examine the cumulative abundance relationships between colonisation days, we were using the ggstatsplot package (Patil 2021). The IndVal analyses were performed with the multipatt function of the ‘indicspecies’ package (seed = 999, perm = 9999) (De Cáceres and Legendre 2009).

The heatmap was created using the ‘ComplexHeatmap’ package (Gu et al. 2016). The taxa displayed on the heatmap were clustered using the UPGMA method and Euclidean distance with the hclust and dist functions of the “stats” package in R Studio. The calculation of colonisation and extinction rates, along with subsequent analyses based on these rates, was carried out using the Island package (Chao et al. 2014; Ontiveros et al. 2019). To assess the peritrich destruction caused by the predator *Trachelius ovum* , the Mann–Whitney test was applied, using the ggbetweenstats function. Functional redundancy was calculated as the mean number of species with non‐zero abundance per functional group, calculated only across functional groups present in the sample.

## 3 Results

### 3.1 Taxonomic Structure of Functional Groups

The aim of the study was to observe the colonisation patterns of colonial peritrichs. Furthermore, we sought to identify protists within the biofilm that either colonise the surfaces of colonial peritrichs or are capable of preying upon them.

**FIGURE 2:**
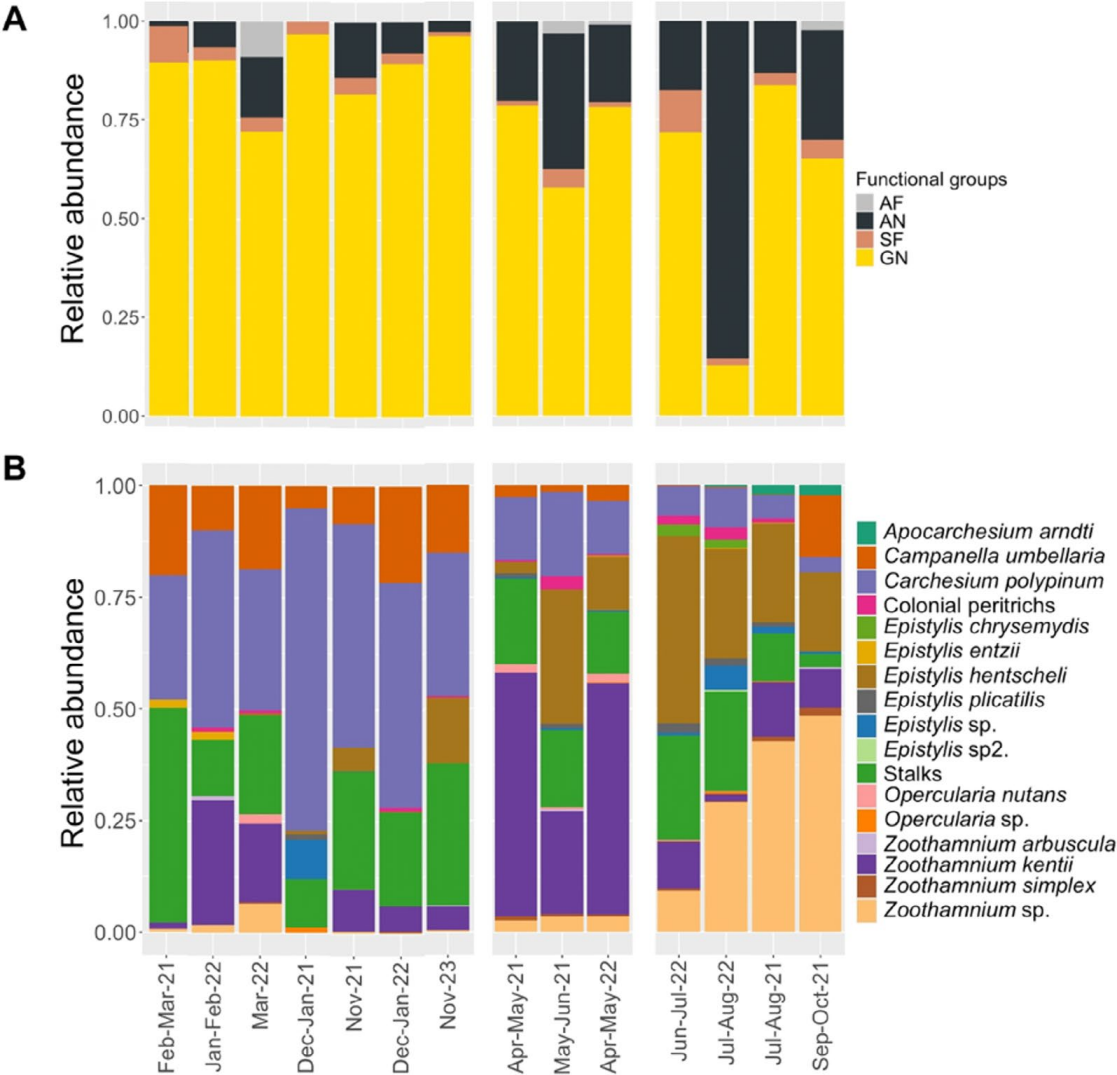
Temporal changes in the relative abundance of functional groups and glass‐attached colonial peritrich taxa throughout the study period. Peritrich colonies were considered as individual entities. Seasonal variation of functional groups (GN, AN, AF, SF) (A). Seasonal patterns in the distribution of GN functional group members (glass‐attached colonial Peritrichia) (B).

Throughout the study period, we identified 16 colonial peritrich taxa (GN functional group) on the deployed glass substrate (Figure 2). One colonial peritrich species (*Epistylis epibioticum*) was not found on the glass surface but instead colonised other colonial peritrichs; this species was therefore assigned to the AN functional group. In addition, we recorded 25 taxa that settled on the stalk structures of various colonial peritrich taxa without feeding on them (AN functional group), as well as 4 taxa that remained closely associated with colonial peritrichs and fed on them using specialised sucking apparatus (suctorial tentacles) (AF functional group). Furthermore, we identified 14 free‐swimming taxa capable of feeding on colonial peritrichs (SF functional group, Table S3).

Among all observed taxa, colonial peritrichs—whether quantified per zooid or per colony—accounted for the largest share of the community's relative abundance (Figure 2). In the summer of 2022, the elevated abundance of epibionts was primarily driven by the proliferation of choanoflagellates, whose abundance markedly increased during this period (Table S3).

### 3.2 The Seasonal Pattern of Sessile Peritrichs and Their Relationship With Environmental Variables

**FIGURE 3:**
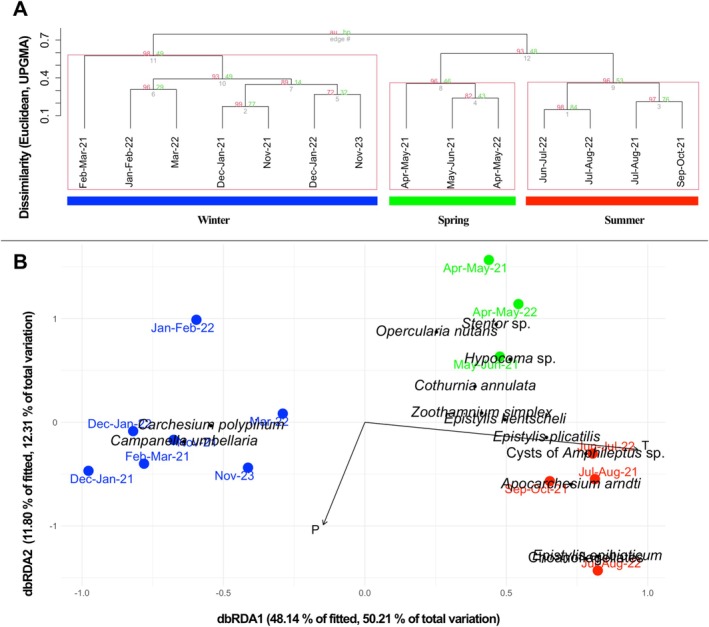
Relationship between colonial peritrich communities and environmental variables. Classification of peritrich samples (Euclidean distance, UPGMA) showing three distinct clusters of glass‐attached peritrich communities across the sampled months (A). dbRDA of community structure versus environmental parameters (B). Peritrichia and all other taxa included, colour coding as in panel (A).

A dendrogram based on the abundance data of sessile peritrichs across 14 study months revealed three main clusters. The large clusters identified based on the relative taxon abundance variables of sessile peritrichs correspond to the seasons; therefore, these will be referred to as winter, spring, and summer samples in the subsequent analyses. In the winter cluster samples, the water temperature was below 12°C, which formed the basis for its designation, despite the fact that certain November samples and one March sample also clustered here (Figure 3A).

### 3.3 Seasonal Fluctuation of Environmental Variables

During the study period, the winter months were characterised by low water temperatures (3.1°C–10.3°C), whereas higher temperatures were typical of spring (13.4°C–20.4°C) and summer (14.9°C–22.5°C). Elevated electrical conductivity (EC: 484.5–614 μS/cm) and mineral nitrogen concentrations (2.1–3.3 mg/L) occurred during winter, while lower values were observed in summer (EC: 360.5–424.8 μS/cm; mineral N: 1.1–1.9 mg/L) and spring (EC: 366.7–451.7 μS/cm; mineral N: 1.3–1.5 mg/L).

The highest dissolved oxygen concentrations were recorded in winter (9.0–11.2 mg/L) and spring (8.5–11.4 mg/L), whereas summer months were characterised by lower values (7.5–8.8 mg/L). In contrast, oxygen saturation was lower in winter (71.3%–93.8%) than in spring (90.7%–115%) and summer (77.6%–100.8%).

The lowest phosphate‐P concentrations were detected in spring (10.2–45 μg/L), while higher concentrations were characteristic of summer (50.0–76.7 μg/L) and winter (20.0–71.6 μg/L).

### 3.4 General Seasonal Pattern

Principal component analysis (PCA) of recorded environmental variables reproduced the three distinct clusters previously identified through hierarchical classification (Figure S2).

Significant environmental variables influencing the protist communities were identified using distance‐based redundancy analysis (dbRDA, Figure 3B). Temperature (forward selection, ANOVA: *F* = 13.0043, Df = 1, AIC = 16.0357, *p* = 0.0001) and phosphate‐P content (forward selection, ANOVA: *F* = 3.9277, Df = 1, AIC = 9.3855, *p* = 0.0143) emerged as the two most influential environmental factors.

In the dbRDA ordination plot, according to the orientation of the temperature vector, the samples representing the members of the winter cluster in the previous hierarchic classification's dendrogram clearly represent cold‐water communities typical of autumn and winter and are distinctly separated from the spring and summer (and some autumn) samples originating from warmer periods. The three groups of the samples were significantly separated from one another (ANOSIM: significance: *p* = 1e−04, *R*: 0.9279). During the study period, abundance peaks typically occurred in spring, but an autumn and a late‐summer peak were also observed (Figure 5).

The genera *Carchesium* and *Campanella* were present with high relative abundance in all seasons, both when quantified per colony (Figure 2) and per zooid (Table S3). In terms of relative abundance, winter biofilms were dominated by *Carchesium* and *Campanella*, whereas spring and summer assemblages were dominated by *Zoothamnium*, *Epistylis*, and *Apocarchesium*.

Seasonal indicator taxa were identified using IndVal analysis and are displayed on the dbRDA plot. The taxa characteristic of the summer period included choanoflagellates, *Apocarchesium arndti*, and *Epistylis epibioticum*, whereas the spring period was characterised by *Opercularia nutans*, *Stentor* sp., and *Hypocoma* sp. Both warm seasons were associated with *Epistylis hentscheli*, *Cothurnia annulata* , *Zoothamnium simplex* , and *Epistylis plicatilis* . In addition, these periods were marked by the presence of *Amphileptus* sp. cysts found on the stalks of colonial peritrichs. The IndVal analysis did not identify any significant indicator taxa for the winter period (Table 1).

**TABLE 1:** Indicator species of different seasons.

	Taxa	IndVal	*p*
Winter	—	—	—
Spring	*Opercularia nutans*	0.910	0.0204
	*Stentor* sp.	0.816	0.0341
	*Hypocoma* sp.	0.796	0.0454
Spring and summer	*Epistylis hentscheli*	0.960	0.0234
	*Cothurnia annulata*	0.954	**0.0059**
	*Zoothamnium simplex*	0.941	**0.0094**
	Cysts of *Amphileptus* sp.	0.925	0.0464
	*Epistylis plicatilis*	0.837	0.0378
Summer	Choanoflagellates	0.983	0.0279
	*Apocarchesium arndti*	0.866	0.0147
	*Epistylis epibioticum*	0.866	0.0152

*Note:* Indicator values (IndVal) represent the relative importance of taxa ranging from 0 to 1. Significance (*p*) levels in bold designate *p* < 0.01.

### 3.5 General Colonisation Patterns

**FIGURE 4:**
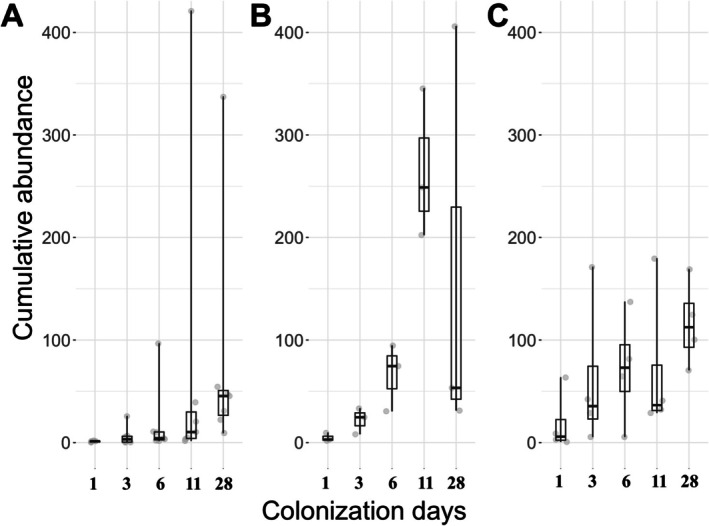
Temporal variations in cumulative abundance in winter (A), spring (B) and summer (C) on days 1, 3, 6, 11 and 28. Peritrich abundance is based on zooid numbers. Choanoflagellates and stalks are excluded.

During winter colonisation, the cumulative biofilm abundance showed an increasing trend throughout the entire colonisation period (Figure 4A). In spring, cumulative abundance declined on day 28 (Figure 4B). In summer, a decrease in cumulative abundance was observed between days 6 and 11 of colonisation; however, by day 28, the abundance exceeded the values recorded on the preceding sampling days.

**FIGURE 5:**
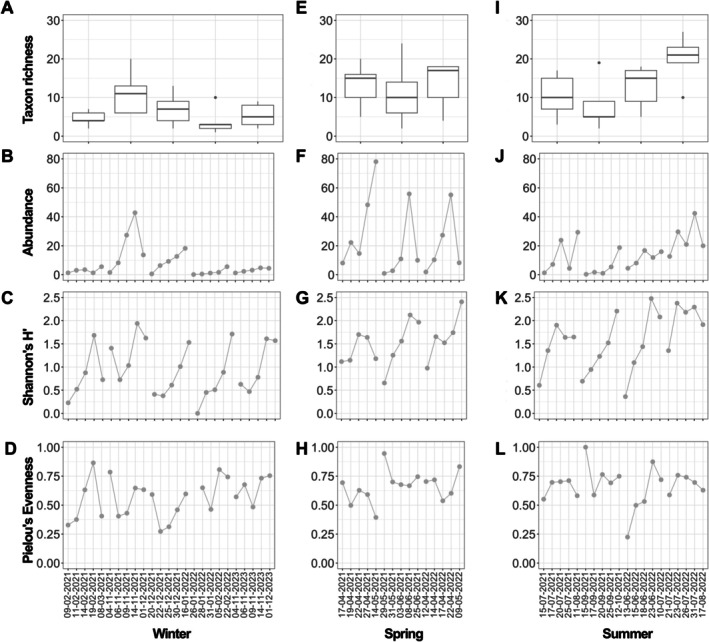
Colonisation curves and species richness of examined communities on artificial substrates by different seasons (A–L). Monthly variations in species richness (A, E, I), temporal variations in abundance (B, F, J), Shannon's H′ diversity index (C, G, K), and Pielou's evenness (D, H, L) during winter, spring and summer. Peritrich abundance based on colony numbers. Choanoflagellates and stalks excluded. Months Mar‐2022 and Dec‐Jan‐2021 not presented, due to the lack of colonial peritrichs on the first colonisation days.

Among the study months, winter colonisation days were generally characterised by lower taxon richness compared with those in summer or spring (Figure 5A,D,G). Similarly, higher Shannon's H′ diversity values were characteristic of spring and summer. In many cases, diversity on the first day of colonisation in spring and summer exceeded that recorded on the sixth day of winter colonisation. Across all seasons, when a decline in Shannon's H′ diversity was observed, it occurred on Day 28 of colonisation (Figure 5B,E,H).

During winter colonisation, evenness exhibited a characteristic pattern in most cases, with the highest values occurring during the early and late stages of colonisation (Figure 5D). A similar pattern was recorded only once during an early summer month (May 29–June 25, 2021).

### 3.6 Indicator Taxa for Different Colonisation Days in Seasonal Resolution

Protist communities on the artificial substrate were further analysed according to colonisation day. In this section, we present the significant indicator taxa for each colonisation day in all seasons (Table 2).

**TABLE 2:** Seasonal indicator species across different colonisation days.

Day	Winter		Spring		Summer	
	Taxon	IndVal	Taxon	IndVal	Taxon	IndVal
3–28	—	—	*Vorticella convallaria* *	0.984	*Zoothamnium* sp.**	0.993
6–28	—	—	Epistylis hentscheli*	0.983	Zoothamnium kentii***	0.984
			*Cothurnia annulata* *	0.939	*Cothurnia annulata* *	0.872
11	*Litonotus cygnus* *	0.701	—	—	—	—
11–28	*Campanella umbellaria* ***	0.947	Opercularia nutans**	0.995	—	—
	*Vorticella convallaria* *	0.766	*Zoothamnium simplex* *	0.913		
28	Amphileptus procerius**	0.756	—	—	Choanoflagellates*	0.967
					*Acanthocystis* sp.*	0.805
					Apocarchesium arndti*	0.799

*Note:* Indicator values (IndVal) represent the relative importance of taxa from 0 to 1. Significance levels are denoted by asterisks: *p < 0.05; **p < 0.01; ***p < 0.001.

For none of the three studied seasons did IndVal analysis identify significant indicator species for Day 1 of colonisation. Likewise, no specific indicator taxa were detected for Days 3 or 6. In contrast, the later colonisation days (Days 11, 28) yielded significant indicators in all seasons.

In winter, the predator *Litonotus cygnus* was characteristic of Day 11 colonisation. Among colonial peritrichs, *Campanella umbellaria* was indicative of Days 11, 28, accompanied by the predator *Amphileptus procerius* and the epibiont *Vorticella convallaria* .

In spring, excluding Day 1, *Vorticella convallaria* was an indicator taxon for all subsequent colonisation days (Days 3, 6, 11, 28). *Epistylis hentscheli* was characteristic of Days 6, 28, accompanied by the epibiont *Cothurnia annulata* . The late colonisation days in spring (Days 11, 28) were characterised by the colonial peritrichs *Opercularia nutans* and *Zoothamnium simplex* .

In summer, excluding Day 1, the colonial peritrich *Zoothamnium* sp. was an indicator for all later colonisation days. *Zoothamnium kenti* and the epibiont *Cothurnia annulata* were indicators of Days 6–28. On Day 28 only, indicators included the colonial peritrich *Apocarchesium arndti*, epibiotic choanoflagellates, and *Acanthocystis* sp.

### 3.7 Functional Groups During Colonisation

**FIGURE 6:**
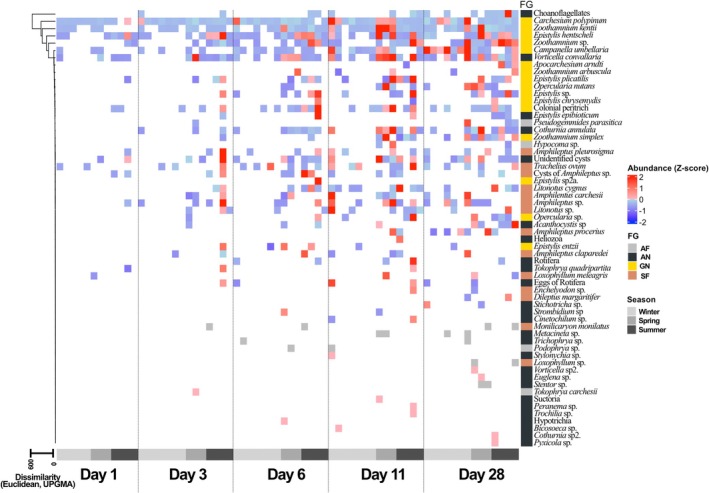
Heatmap of the absolute abundance of functionally categorised taxa by colonisation day. Taxa are ordered according to hierarchical clustering (UPGMA, Euclidean distance) based on their absolute abundance values over the entire study period.

The colonisation process exhibited a similar pattern of functional group establishment across all three seasons. On the first day of colonisation, colonial peritrichs (GN) settled on the glass slides alongside potentially predatory, free‐swimming taxa (SF) and non‐predatory epibionts that colonised their surfaces (AN) (Figure 6). At this stage, only three taxa exceeded their average abundance: the colonial peritrichs *Carchesium polypinum* and *Epistylis hentscheli* (GN), and unidentified cysts (AN).

As colonisation progressed, functional groups that initially contained only one or two species with below‐average abundances became enriched with additional taxa, resulting in diverse, functionally redundant communities by the later colonisation days in all seasons.

On Day 3, several SF taxa (*Amphileptus* spp., *Litonotus* spp.) exceeded their average abundances, and the AF group—species feeding on colonial peritrichs via suctorial structures—appeared for the first time, represented solely by *Tokophrya carchesii*. In the AN group, *Vorticella convallaria* was recorded with above‐average abundance.

By Day 6, the functional groups expanded further with additional species. The AF group was represented only sporadically, by a single taxon (*Podophrya* sp.). Species of the AF group—predatory, suctorial feeders closely associated with colonial peritrichs—reached above‐average abundances only during the later colonisation period (Days 11–28).

The AF group was particularly characteristic of the spring and summer periods. It is important to highlight that the proportion of epibionts (AN) relative to colonial peritrichs (GN) showed a significant correlation with the average water temperature changes over the months examined (linear regression: *R* ^2^ = 0.5214, *p* = 0.003541), even when mass choanoflagellate occurrences in summer were excluded from the analysis (Figure S3).

The general pattern observed during the colonisation Days 1–28 was that higher‐than‐average abundances were typical of the spring and summer periods across most functional groups and taxa (Figure 6, see horizontal arrangement of samples).

An exception to this was observed during the winter period on colonisation Days 11–28, where the GN group appeared with abundances exceeding the average, which was largely attributable to the species *Campanella umbellaria* . Additionally, on colonisation Day 11 in winter, members of the SF group, particularly *Amphileptus carchesii* and *Trachelius ovum* , exhibited abundances notably higher than average.

**FIGURE 7:**
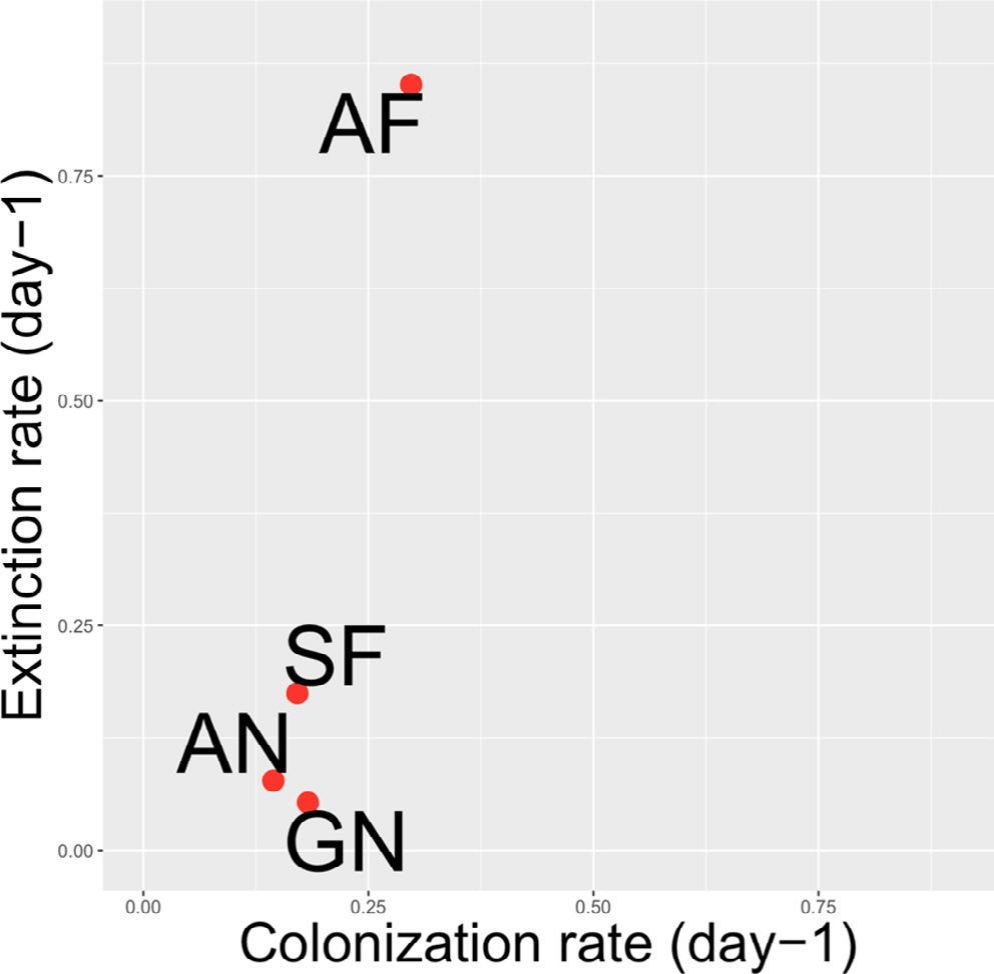
Colonisation and extinction rates of combined functional groups over the study period. GN—glass‐attached sessile non‐peritrich feeders; SF—free‐swimming potential peritrich feeders; AN—crawling, peritrich‐attached non‐feeders; AF—crawling, peritrich‐attached feeders with prolonged prey attachment. Corresponding taxa for each functional group are listed in Table S3.

Among the functional groups, the AF group exhibited distinct colonisation characteristics compared to the others. The AF group had the highest colonisation and extinction rates (Figure 7). The colonisation characteristics of the SF groups (those feeding facultatively or obligately on peritrichs) were similar to GN and AN. The GN and AN groups showed similar colonisation rates to SF but were characterised by lower extinction rates.

Thus, diverse and functionally redundant communities were established. As colonisation progressed, the various functional groups (GN, AN, SF, AN) expanded with the addition of new taxa, resulting in high functional redundancy during the later colonisation days (11–28). This phenomenon was particularly pronounced during the spring and summer months.

### 3.8 Interactions Among Species

During the hierarchical clustering of species (Figure 6, displayed to the left of the heat map), those frequently found on the surfaces of colonial peritrichs—specifically species from the AN group (e.g., *Vorticella convallaria* )—and species from the AF group (e.g., *Hypocoma* sp., *Pseudogemmides parasitica*), which occur only sporadically but in large numbers and feed on colonial peritrichs, clustered closely together. Taxa belonging to the SF group, which potentially feed on peritrichs, clustered further away from them. The species with low abundance that only occasionally occur on the surface, belonging to the AN functional group, formed the most distant cluster from the colonial peritrichs (Figure 6).

**FIGURE 8:**
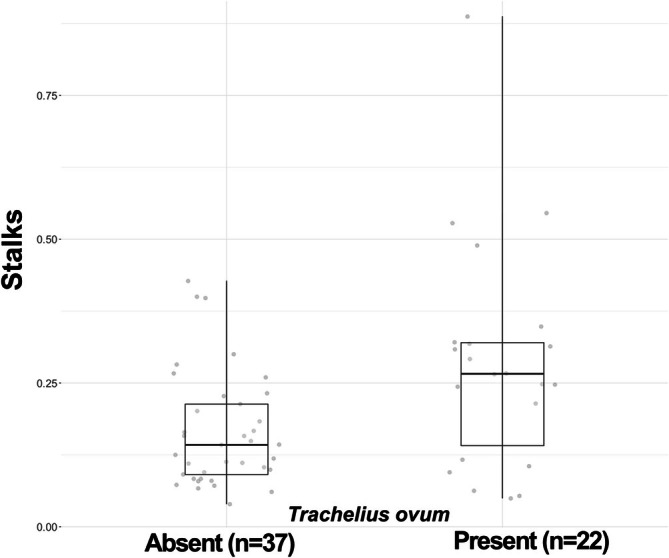
The impact of the predatory ciliate *Trachelius ovum* on the colonial peritrich community: The ordinate label ‘Stalks’ denotes the number of stalks originating from deceased peritrichs.

*Trachelius ovum* is a key member of the studied biofilm community within the SF functional group during all colonisation days (Figure 8). In the presence of *Trachelius ovum* , the proportion of stalk remnants relative to live colonial peritrichs was significantly higher, a phenomenon attributed to the predation by *Trachelius ovum* .

## 4 Discussion

### 4.1 Community Composition and Seasonal Dynamics of Peritrichs in River Biofilms

The ecological significance of peritrichs in river biofilms is underscored by their particularly high mean monthly abundance in older biofilms, as demonstrated in a previous study on artificial glass substrates in the Rhine (Ackermann et al. 2011). The present study focused exclusively on peritrich ciliates and their associated heterotrophic microeukaryotes, including attached heterotrophic flagellates (above 7 μm) and free‐swimming ciliates, and other conspicuous heterotrophic protists (e.g., *Acanthocystis* sp.), as well as rotifers. Similar to the experimental study by Ackermann et al. (2011), almost the entire abundance of heterotrophic flagellates was made up of choanoflagellates. In contrast to the mature biofilms of the Rhine (Ackermann et al. 2011), where algae became dominant after approx. 1 month, it can be concluded that during the short‐term colonisation periods in the Danube, algae were not present in sufficient abundance to impede the examination of peritrichs within the biofilm.

#### 4.1.1 Seasonal Dynamics of Colonial Peritrichs

Studies on running water environments are scarce, with most focusing on short‐term faunistic surveys or environmental condition assessments and impact studies (Scherwass et al. 2010; Tan et al. 2010; Babko et al. 2016; Pliashechnyk et al. 2018; Bykova 2021). There is even less data available on the seasonal dynamics of heterotrophic protists in lotic environments. Furthermore, the majority of existing data pertains to the seasonal patterns of planktonic communities (Nosek and Bereczky 1994; Lair et al. 1999; Scherwass and Arndt 2005; Kiss et al. 2009; Pauleto et al. 2009; Rakshit et al. 2014; Wu et al. 2018; Cruaud et al. 2019; Zhang et al. 2022). In lotic environments, ciliate communities exhibit distinct seasonal patterns, typically characterised by a summer minimum and a spring or autumn maximum (Blatterer 2008). In the Soroksár Branch of the Danube, algal abundance peaks have been reported in spring, autumn, and winter (Vadadi‐Fülöp et al. 2007). Long‐term algological records from the middle section of the Danube suggest a shift toward oligotrophication (Abonyi et al. 2018, 2020), but these studies did not encompass the Soroksár Branch, and our investigation likewise did not address this slow‐flowing section in this context. Seasonal dynamics of heterotrophic protists have not previously been examined in the Soroksár Danube Branch.

During the study period, the biofilm was characterised by spring and late summer and autumn abundance peaks. The spring peak was likely driven by the seasonally increasing availability of planktonic algae and bacteria. A similar spring peak in biomass was observed in the River Rhine by Kathol et al. (2011), while the co‐occurrence of spring and autumn biomass peaks has been reported from lakes in Poland (Mieczan 2010). The autumn abundance peak observed in November 2021 in our study was probably associated with increased bacterial availability resulting from the decomposition of organic matter entering the water during leaf fall (Blatterer 2008; Lang et al. 2015). Furthermore, in November 2021, the development of the peritrich biofilm during the colder autumn period may have been facilitated by the reduced activity of invertebrates feeding on the biofilm (Jacoby 1987; Beck et al. 2019). The lowest abundance values were recorded in the distinctly winter months (December, January, February). Another seasonal pattern was that spring and summer were characterised by higher species richness and higher Shannon's H' diversity values compared with the winter period.

In our study, the autumn–winter, spring and summer protist communities were clearly distinct from one another (Figure 3; db‐RDA, ANOSIM results). Winter biofilms were particularly dominated by *Carchesium polypinum* and *Campanella umbellaria* . However, as these species were also common in other seasons, they were not identified as winter indicator species in the IndVal analysis.

In spring, *Zoothamnium kentii* was the most dominant colonial peritrich, although it was abundant throughout the year. The indicator species for the spring period was *Opercularia nutans*. During summer, *Zoothamnium* spp., *Epistylis* spp. and *Apocarchesium arndti* became dominant, with *Apocarchesium arndti* and *Epistylis epibioticum* identified as indicator species for this season. A pattern very similar to our observations was reported by Kathol et al. (2011), who attributed it to the trophic properties of the species: bacterivorous species (e.g., *Campanella umbellaria* , *Carchesium polypinum* ) being more common in winter, while species capable of algivory (e.g., *Zoothamnium* spp., *Epistylis* spp.) dominated in summer. However, based on our own observations and literature data, we found that *Campanella umbellaria* also feeds on algae, while several *Zoothamnium* species are primarily bacterivorous (Foissner et al. 1992).

We believe that the seasonal pattern of colonial peritrichs is primarily driven by the physical and chemical parameters of the water. Among the environmental variables examined, water temperature and phosphate‐P significantly explained the differences in protist community composition. The significant influence of water temperature on biofilms has been demonstrated by several authors (Arndt et al. 2003; Gong et al. 2005; Kathol et al. 2009; Norf et al. 2007; Ackermann et al. 2011; Wey et al. 2012; Marcus et al. 2014; Zhao et al. 2023). However, there are exceptions to this pattern. In a lentic environment, Pratt et al. (1986) did not observe a significant effect of temperature on species richness, although they suggested that temperature could potentially influence colonisation rates. Marcus et al. (2014) found that in disturbed, temperature‐limited environments (winter), an increase in water temperature enhanced the dominance of peritrichs in the biofilm, leading to a reduction in overall biofilm diversity. In our diversity analyses, we did not distinguish between peritrich and non‐peritrich components of the biofilm; however, in future studies encompassing the entire biofilm community, such differentiation would be worth pursuing.

### 4.2 Changes in Peritrich Ciliate Assemblages During Colonisation

The formation of biofilms can be divided into the following stages: lag, exponential growth, decreasing rate, plateau and sloughing (Bryers and Characklis 1982). However, for clarity, in this paper, we will use the terms ‘colonisation’, ‘peak’, and ‘decline’, following Harmsworth and Sleigh (1993). These terms are, in our opinion, highly applicable to describe changes in abundance dynamics.

In winter, the cumulative abundance of the biofilms increased steadily over successive colonisation days, suggesting that for biofilms characterised by lower colonisation rates (species/day), only the colonisation and peak phases were captured (Figure 4A). In spring, cumulative abundance showed a slight decline by the 28th colonisation day, indicating that this period likely corresponded to the decline phase of colonisation (Figure 4B). In summer biofilms, which exhibited the highest colonisation rates, a decrease in cumulative abundance was already apparent by day 11. We therefore assume that in the warmer environment the decline phase occurred earlier (Figure 4C).

**FIGURE 9:**
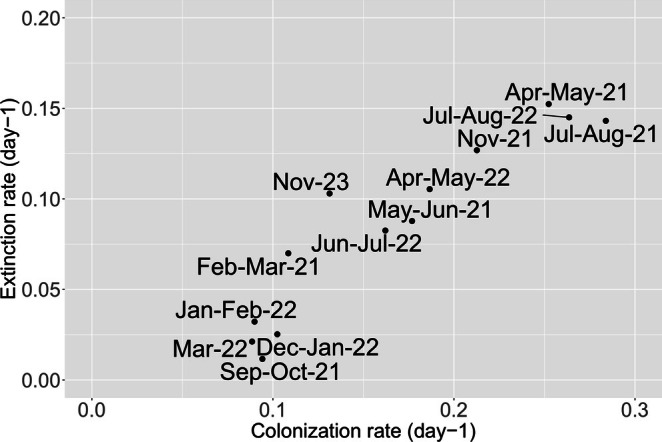
Colonisation and extinction rates of communities across sampling months. The December 2020–January 2021 period, likely affected by undersampling and lower winter abundances, is excluded as an outlier.

In terms of taxon turnover rates, the warm summer and spring months exhibited the highest dynamics, characterised by the greatest colonisation and extinction rates, whereas biofilms during the colder winter months showed less dynamic changes (Figure 9). A similar pattern, featuring slower autumn and faster summer colonisation, was reported by Pratt et al. (1987) in their colonisation study of the Flint River. Villanueva et al. (2010) observed faster colonisation even with a 3°C temperature increase, during which peritrichs constituted one of the most significant groups in the community.

#### 4.2.1 Dominance Dynamics During Colonisation

Due to the slower colonisation during the cold period, a more detailed colonisation pattern was observed (Figure 5C). During these months, evenness displayed a characteristic ‘U’‐shaped trajectory. On Days 1–3, few species with low abundances were present. Because abundances were uniformly low, variation among species was minimal, resulting in high evenness (represented by the upper‐left portion of the ‘U’). Between Days 3 and 6, one or two species from the initial pool proliferated substantially, while additional colonisers appeared at low abundances. During this phase of colonisation, evenness declines as one or two species become markedly dominant (corresponding to the bottom of the ‘U’). After Day 6, the community diversified with the addition of further species, while the dominance of the initially prevalent species diminished and less abundant species increased in relative abundance. This results in an increase in evenness (represented by the right arm of the ‘U’). The observed fine‐scale pattern suggests an initial random settlement of seasonally characteristic species, followed by selection for more efficient colonisers.

It is important to highlight that IndVal analysis of the colonisation days, conducted separately for each season, did not identify indicator species for the 1‐day‐old biofilms. This result remained consistent even when the IndVal analysis was run on the entire dataset without seasonal separation. Furthermore, during the slower winter colonisation, indicator species were only detected on Days 11 and 28, whereas in spring and summer, indicator species were identified from Days 3 to 28. We interpret the absence of early winter indicator species as evidence of random settlement by taxa from the seasonally characteristic species pool during early colonisation. Due to this random occupation of the taxon pool by different taxa in each season, we suggest that biofilm formation is best explained by the lottery model of community assembly proposed by Chesson and Warner (1981).

In spring and summer, we were able to identify indicator species for Days 3–28. These included not only colonial peritrichs but also their epibionts, such as *Vorticella convallaria* —which is strongly attached to the stalks of colonies (Days 3–28, spring)—and *Cothurnia annulata* (Days 6–28, summer and spring). Indicator species of the 28‐day summer biofilms also included choanoflagellates settling on the colony surfaces and *Acanthocystis* sp. This is consistent with the study by Leadbeater (2015), which suggests that choanoflagellates may be particularly successful during late colonisation.

Our observations suggest that the late colonisation phase may be better explained by the physical ecosystem engineering model proposed by Jones et al. (2010), rather than the lottery model of Chesson and Warner (1981). However, both hypotheses warrant further investigation in future studies.

### 4.3 Biotic Interactions and Their Inferred Roles

Pratt and Cairns Jr (1985) primarily emphasise biotic interactions in the context of colonisation monitoring, dividing the process into two phases: non‐interactive and interactive. The transition between these phases is marked by the establishment of an equilibrium species richness. However, our observations indicate that even the earliest colonisation days were not free of interactions. Notably, peritrich predation was already evident on Day 1, with large ciliated predators such as *Trachelius ovum* actively consuming them. Furthermore, cysts of *Amphileptus* spp., which feed on peritrichs, were found on the stalks of colonial peritrichs settling on the glass slides. On the first colonisation days, non‐predatory protists such as *Vorticella convallaria* and *Tokophrya quadripartita* also appeared on the stalks of colonial peritrichs (Figure 6). Therefore, it is important to emphasise that the early colonisation phase cannot be classified as non‐interactive, as suggested by Pratt and Cairns Jr (1985). Nonetheless, we support the idea that biotic interactions intensify as colonisation progresses (Figure 6).

#### 4.3.1 Trophic Interaction Between Taxa: An Example of a Particularly Important Predatory Protist of Colonial Peritrichs

We observed numerous taxa feeding on colonial peritrichs within the AF and SF functional groups (AF: *Hypocompa* sp., *Pseudogemmides parasiticus*; SF: *Amphileptus* spp., *Trachelius* sp.), many of which were particularly abundant. Among these, we focused on the impact of *Trachelius ovum* , notable for its large cell size and high abundance, on the biofilm structure. We found that in the presence of this species, the proportion of deceased peritrich stalk remains within the biofilm increased significantly. *Trachelius ovum* has previously been shown to be capable of reducing the abundance of colonial peritrichs such as *Carchesium polypinum* in biofilms (Nusch 1975). Nosek and Bereczky (1983) also reported a noticeable increase in the number of peritrichs dying within the biofilm in the presence of *Trachelius ovum* , although their results were not quantified. Since *Trachelius ovum* was present during the early colonisation days, we suggest that it may have influenced the subsequent development of the biofilm. However, this hypothesis requires confirmation through future research.

#### 4.3.2 Peritrichs as Ecosystem Engineers

Peritrichs have been widely documented as epibionts of macroinvertebrates (Nenninger 1948; Matthes 1950, 1982; Stiller 1971; Schödel 2018; Safi et al. 2022). However, there is considerably less information available regarding the eukaryotes that colonise peritrichs. The available information is fragmented, and we provide a brief summary below. In his book Animalcula Infusoria (Müller 1786), Müller, in Plate XLVI, fig. 9, illustrated the stalk of a colonial peritrich that was apparently colonised—most likely by numerous choanoflagellates. However, he did not recognise this phenomenon at that time and instead interpreted the epibionts as part of the fine structure of the colony's stalk (‘Squamulae adhaerentes’). Kent had already observed that the stalks of colonial peritrichs are occasionally colonised by choanoflagellates (in Kent 1881, Plate XXXVIII, fig. 6). In 1948, Nenninger reported an unusual phenomenon, the colonisation of the stalk of *Epistylis bimarginata* by *Scyphidia hirudineorum*, a case not previously observed. Matthes (1982) documented *Epistylis apiosomae* occupying *Apiosoma cotti*. Matthes also described a similar association between *Epistylis lwoffi* and *Apiosoma amoebae* (Matthes 1982). Stiller further observed that *Epistylis balatonica* commonly colonised the stalks of *Epistylis entzii* (Stiller 1971). A very similar species in terms of morphology and lifestyle, *Epistylis epibioticum*, was described by Banina (1983). Foissner and colleagues, in a remarkable contribution, shared their observations and bibliographic data on eukaryotes found on peritrichs. They presented microscopic images of *Vorticella* sp. colonising *Zoothamnium kentii* and *Carchesium polypinum* . A shared characteristic of the aforementioned studies is their emphasis on the colonial peritrichs themselves rather than the microeukaryotes colonising them, with the described phenomena primarily presented as notable curiosities. To date, however, no study has explored peritrichs as organisms capable of being colonised by microeukaryotes or examined their role as ecosystem engineers. Based on the results, we propose that colonial peritrichs serve as important ecosystem engineers in biofilms by promoting the emergence of diverse ecological functional groups. The ability of ciliates to act as ecosystem engineers has only recently been proposed (Weerman et al. 2011; Danovaro et al. 2024); however, such a role has not previously been attributed to peritrichs.

In several of Cairns' studies, it is hypothesized that during the ‘interactive’ phase of colonisation, when the equilibrium species richness is reached, the biotic interactions between species are influenced by the extracellular products of various protists (Cairns Jr et al. 1969; Cairns and McCormick 1993). While Cairns' research acknowledges the presence of structures left by protists (e.g., houses), these structures are not quantified (Pratt and Cairns Jr 1985), and their role in the community is not thoroughly discussed. We propose that colonial peritrichs, through both their living presence and the structures they generate, play a significant role in facilitating biotic interactions within the biofilm (Figures 6 and 8).

#### 4.3.3 The Ecological Role of Functional Groups

The proportion of epibionts relative to colonial peritrichs exhibited a significant relationship with the average water temperature changes over the examined months. This suggests that during the warmer periods of the year, the ecological role of colonial peritrichs extends beyond their filtering nutrition, facilitating the connection between planktonic and benthic communities. On the surface of colonial peritrichs, protists, representing additional functional groups, are able to colonise, thereby enriching the functional diversity of the biofilm. During the warmest period, the stalks of peritrichs are heavily colonised by AN. Almost the entire abundance of the AN group consisted of filter‐feeding choanoflagellates, *Vorticella* sp. and *Cothurnia* species. The mass presence of these epibionts can significantly increase the filtering potential of the biofilm. It is important to note that choanoflagellates are ecologically significant filter feeders (Boenigk and Arndt 2002), of which small size also results in a rapid turnover rate (Fenchel and Finlay 1983). However, the AN group was also slightly enriched by species that are predatory but do not consume colonial peritrichs either (e.g., *Tokophrya quadripartita*, *Trichophrya* sp.). In addition to the AN group, we also observed that species from the SF group, which feed on colonial peritrichs, also consume vorticellas that have settled on peritrich colonies. Therefore, the role of the AN group is particularly important, as their presence can facilitate the appearance of other taxa that feed on them. In cases of strong dominance by colonial peritrichs, a decrease in biofilm diversity was observed by Marcus et al. (2014). In such cases, it is essential to conduct both qualitative and quantitative investigations of the epibionts of peritrichs, as a potentially increasing diversity may remain undetected if epibionts are not taken into account.

It is also important to highlight that functional redundancy exhibited an increasing trend throughout the colonisation days in all seasons (Figure S4). The highest values of functional diversity and functional redundancy were characteristic of the warmer spring and summer months. According to the literature, functional redundancy can optimally enhance community resilience against external disturbances (Weisse 2017; Hoppe et al. 2017; Biggs et al. 2020).

Our results suggest that colonial peritrichs enhance the functional diversity and redundancy of biofilms by facilitating the colonisation of taxa that are physically or trophically associated with them. We propose that this latter process may increase the biofilms' resistance to environmental stressors; however, this hypothesis requires further validation through future research.

## 5 Conclusion

Biofilms in warmer environments are considerably more dynamic than those in colder months, characterised by high extinction and colonisation rates.

Owing to their structural diversity and the extensive surface area provided by their stalks, peritrichs promote the establishment of new functional groups within the biofilm via the organisms that colonise them. Our results indicate that examining peritrich epibionts is particularly relevant in warmer environments, where epibiont proportions are higher than in colder conditions.

Peritrichs, through the filter‐feeding organisms that inhabit their surface (choanoflagellates, other peritrichs), greatly increase the surface area available for filtration within the biofilm, a factor that would be worth quantifying in future studies. However, the peritrich‐dominated biofilm harbours not only filter feeders. Predatory ciliates, such as *Trachelius ovum* observed in this study, can substantially modify the three‐dimensional structure of the biofilm by consuming colonial peritrichs.

In the later stages of colonisation, especially under warmer environmental conditions, it will be important to investigate the role of functional redundancy in the resilience of the biofilm community, particularly in the context of ongoing climate change.

## Author Contributions

**Álmos Becz:** conceptualisation, methodology, field work, microscopy, statistical analyses, writing – original draft, review and editing. **Júlia Katalin Török:** conceptualisation, investigation, writing – original draft, review and editing.

## Conflicts of Interest

The authors declare no conflicts of interest.

## Supporting information


**Figure S1:** Schematic illustration of the sampler, including the anchoring system for the glass slides. Quadrats A–D were designated during microscopic examination.

**Figure S2:** Changes in functional redundancy during the progression of colonisation.

**Figure S3:** Relationship between epibionts and water temperature (linear regression).

**Figure S4:** Principal component analysis (PCA) of 14 sampling occasions based on 8 environmental variables (COD, chemical oxygen demand; EC, electric conductivity; N, mineral nitrogen forms; P, reactive phosphor; T, temperature).

**Table S1:** Missing quadrats (marked in red)—quadrats excluded from analyses due to technical issues.

**Table S2:** Averaged background variables used in subsequent analyses.

**Table S3:** Abundance of all taxa over time, categorised by functional groups. Taxa were assigned to functional groups based on the cited literature. Peritrichs were quantified by zooid counts, and functional groups were defined according to protist trophic traits and spatial positioning within the biofilm.

## Data Availability

The data that support the findings of this study are available in the Supporting Information of this article.
