# Supplementary figures and images for: Peritrichs (Ciliophora, Peritrichia) in the Danube: Keystone Organisms in the Formation of Diverse Protist Biofilms
Source: Environ Microbiol Rep. 2025 Oct 15;17(5):e70215. doi: 10.1111/1758-2229.70215 (PMC12527821; doi:10.1111/1758-2229.70215)

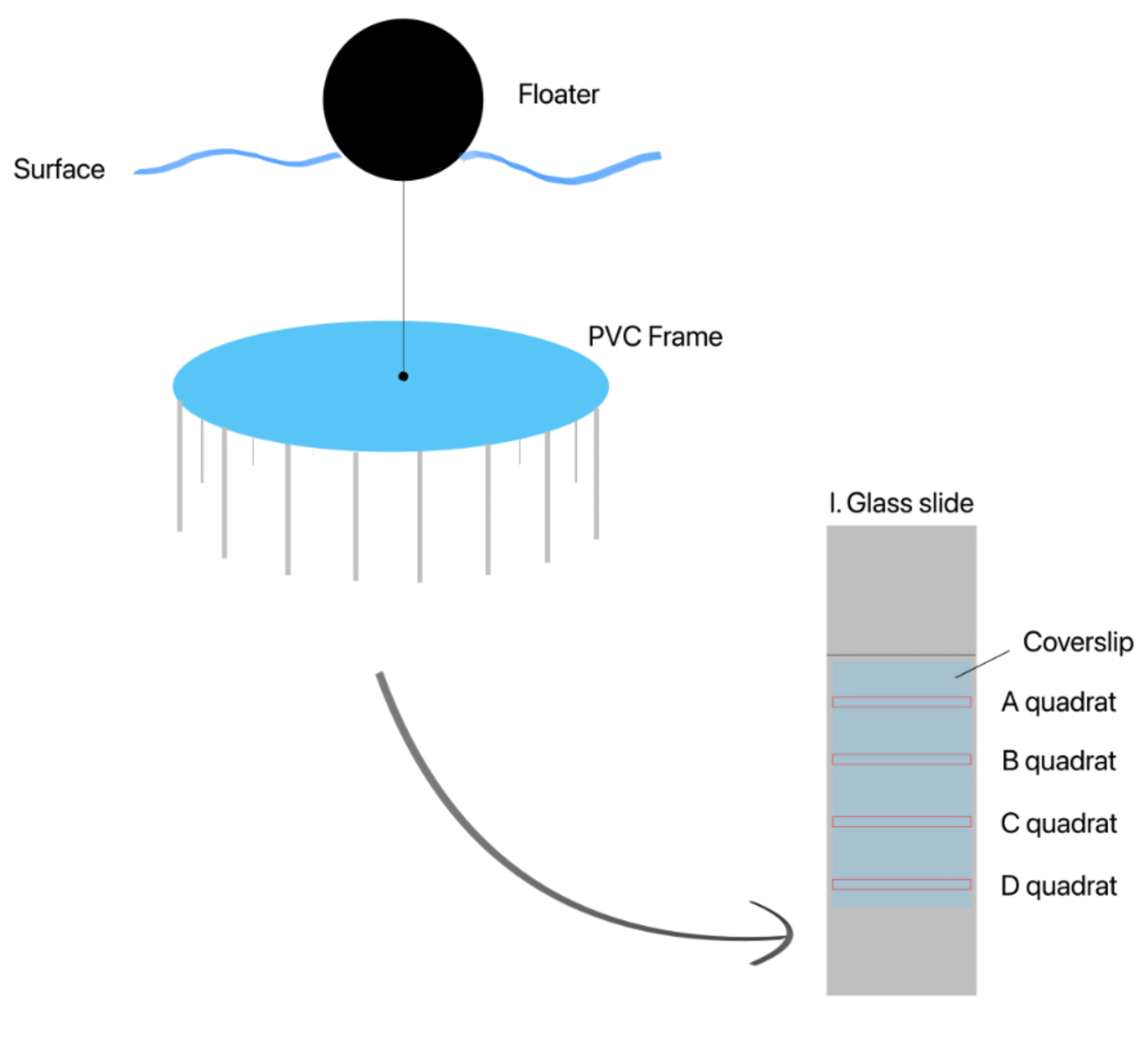

Supplement: Supplementary file 1 — Figure S1: Schematic illustration of the sampler, including the anchoring system for the glass slides. Quadrats A–D were designated during microscopic examination. [file EMI4-17-e70215-s004.tif]

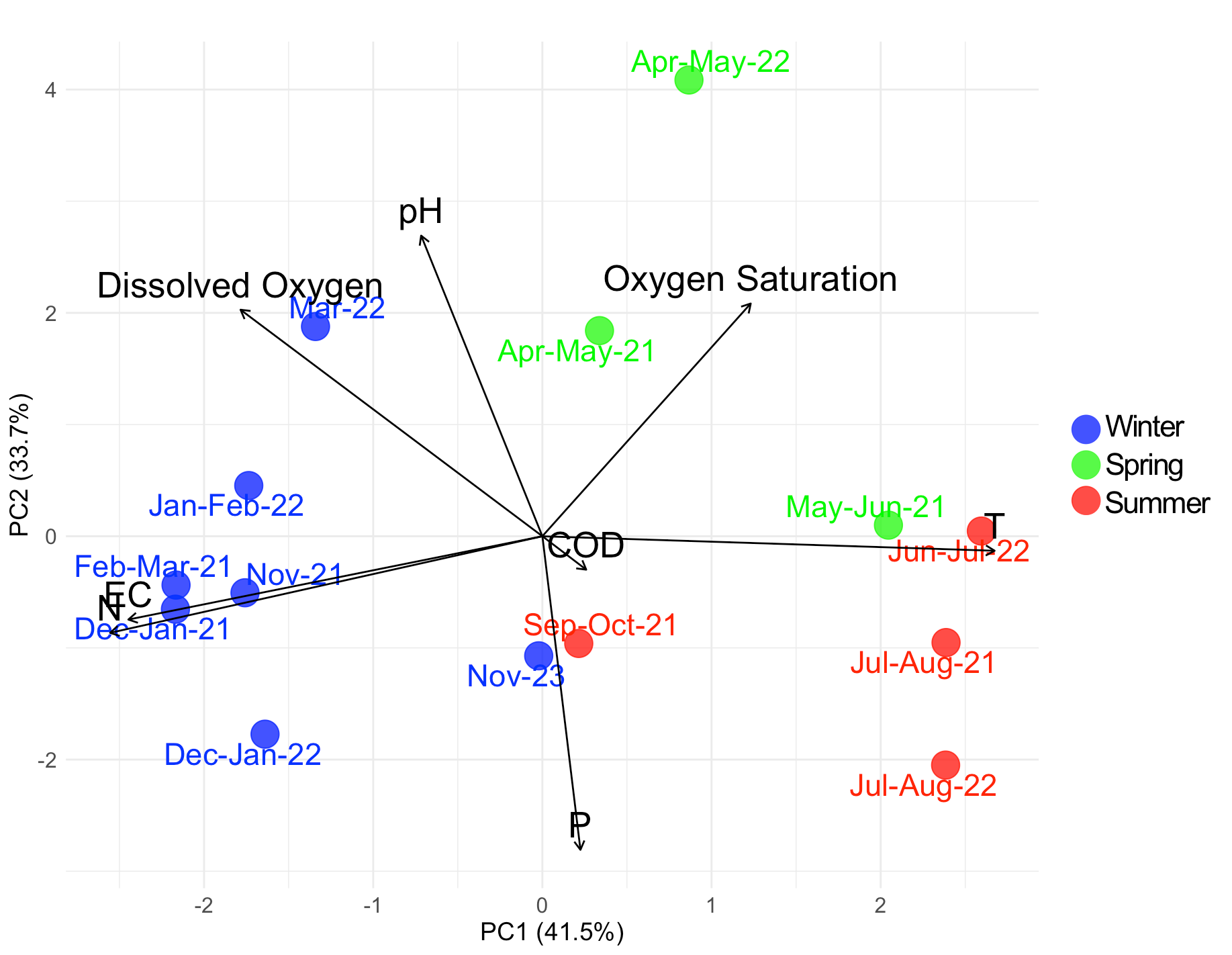

Supplement: Supplementary file 2 — Figure S2: Changes in functional redundancy during the progression of colonisation. [file EMI4-17-e70215-s003.tif]

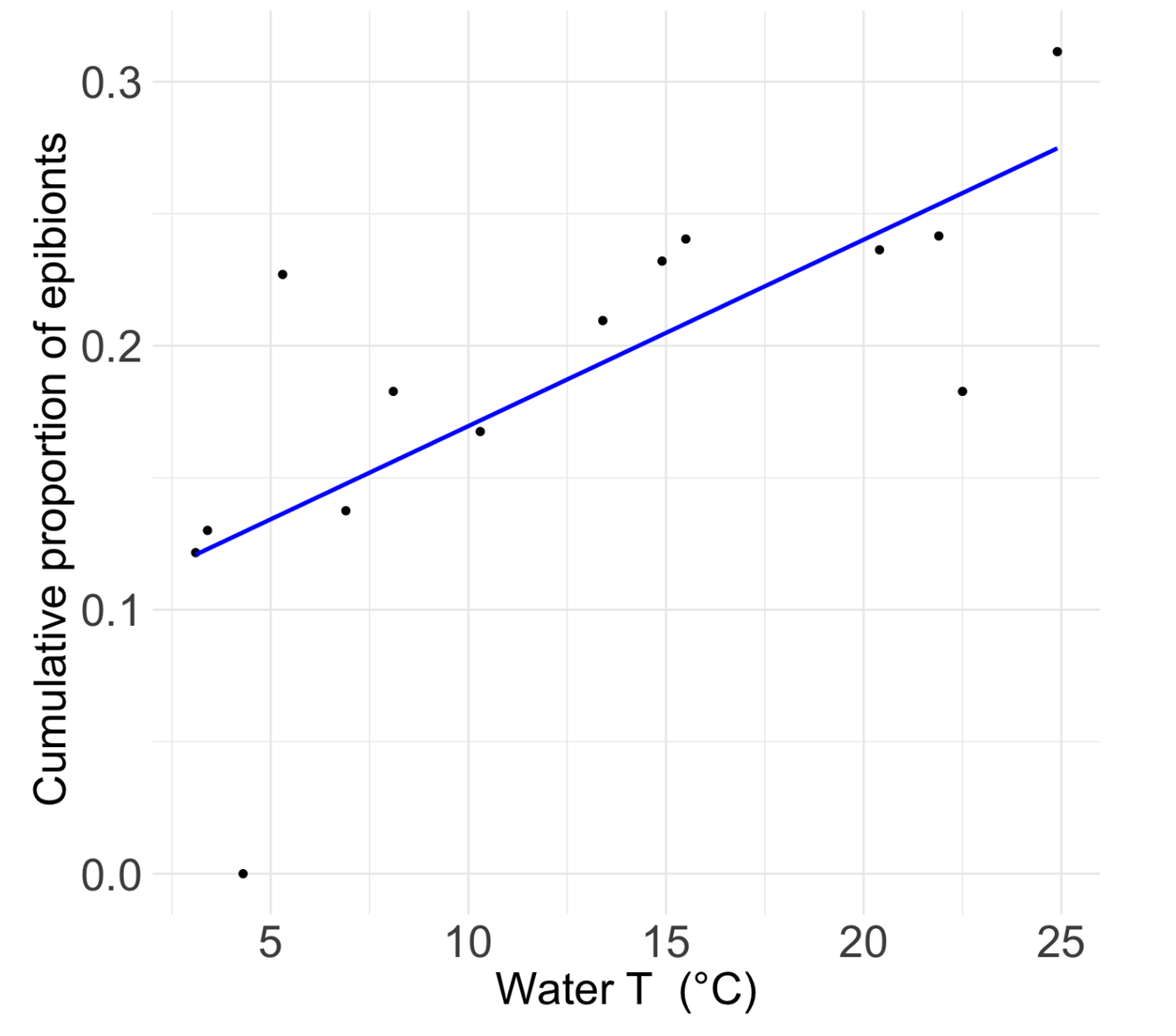

Supplement: Supplementary file 3 — Figure S3: Relationship between epibionts and water temperature (linear regression). [file EMI4-17-e70215-s007.tif]

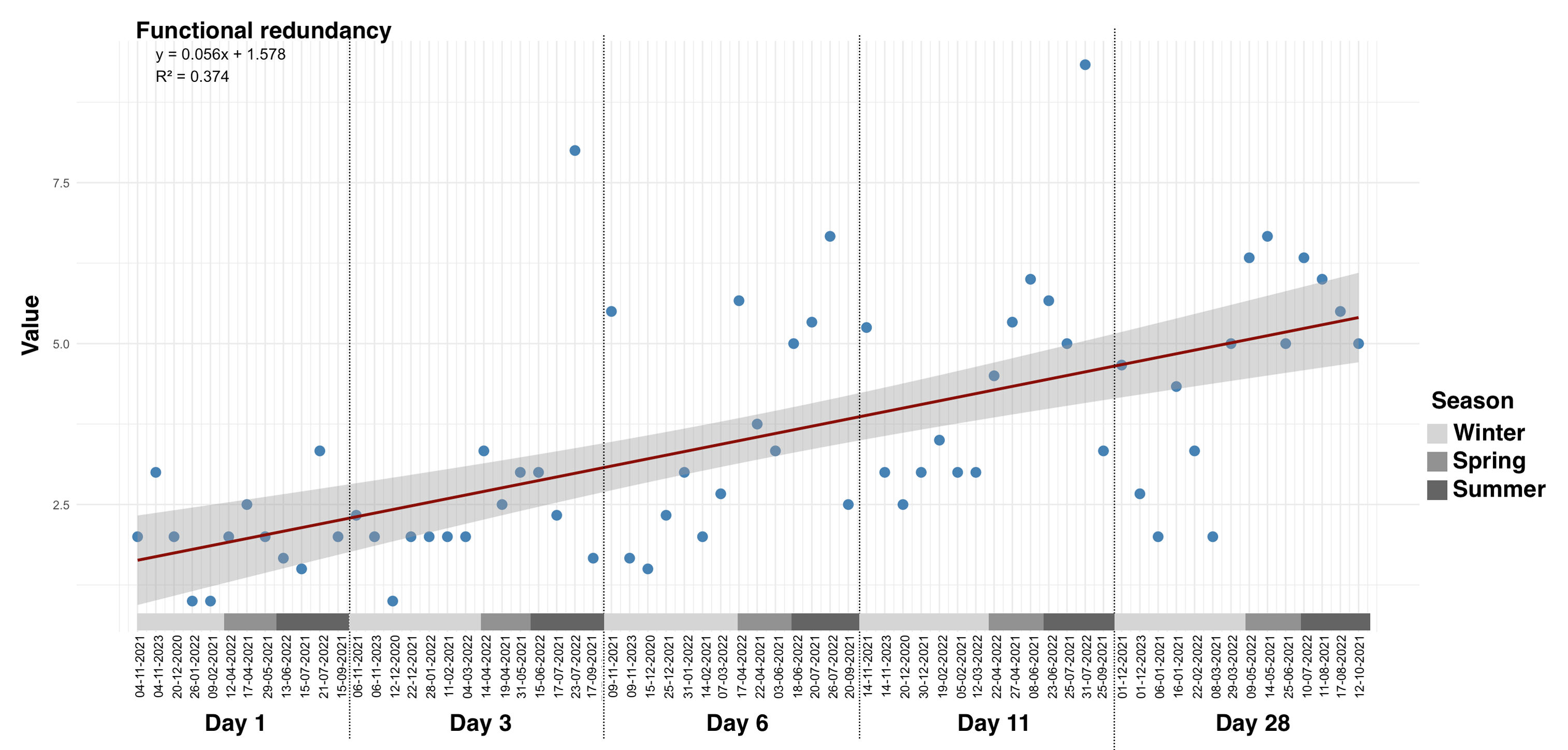

Supplement: Supplementary file 4 — Figure S4: Principal component analysis (PCA) of 14 sampling occasions based on 8 environmental variables (COD, chemical oxygen demand; EC, electric conductivity; N, mineral nitrogen forms; P, reactive phosphor; T, temperature). [file EMI4-17-e70215-s001.tif]
