# Supplementary material for: Peritrichs (Ciliophora, Peritrichia) in the Danube: Keystone Organisms in the Formation of Diverse Protist Biofilms
Source: Environ Microbiol Rep. 2025 Oct 15;17(5):e70215. doi: 10.1111/1758-2229.70215 (PMC12527821; doi:10.1111/1758-2229.70215)
Supplement: Supplementary file 5 — Table S1: Missing quadrats (marked in red)—quadrats excluded from analyses due to technical issues. [file EMI4-17-e70215-s002.docx]

**Table S1.** Missing quadrats (marked in red) — quadrats excluded from analyses due to technical issues.

| Period | Date | Day | I/A | I/B | I/C | I/D | II/A | II/B | II/C | II/D | III/A | III/B | III/C | III/D |
| --- | --- | --- | --- | --- | --- | --- | --- | --- | --- | --- | --- | --- | --- | --- |
| Dec-Jan-2021 | 20.12.2020 | 11. | ok | ok | ok | ok | ok | ok | NA | NA | NA | NA | NA | NA |
| Dec-Jan-2021 | 06.01.2021 | 28. | ok | ok | NA | NA | ok | NA | NA | NA | NA | NA | NA | NA |
| Feb-Mar-2021 | 08.03.2021 | 28. | ok | ok | ok | ok | ok | ok | ok | ok | ok | NA | NA | NA |
| Apr-May-2021 | 27.04.2021 | 11. | ok | ok | ok | ok | ok | ok | ok | ok | ok | ok | ok | NA |
