# Supplementary material for: Peritrichs (Ciliophora, Peritrichia) in the Danube: Keystone Organisms in the Formation of Diverse Protist Biofilms
Source: Environ Microbiol Rep. 2025 Oct 15;17(5):e70215. doi: 10.1111/1758-2229.70215 (PMC12527821; doi:10.1111/1758-2229.70215)
Supplement: Supplementary file 6 — Table S2: Averaged background variables used in subsequent analyses. [file EMI4-17-e70215-s005.docx]

**Table S2.** Averaged background variables used in subsequent analyses.

| **Recording time of environmental variables** | | | **Average values of environmental variables** | | | | | | | |
| --- | --- | --- | --- | --- | --- | --- | --- | --- | --- | --- |
| Dunaharaszti, Szigethalom | Dunaharaszti, Szigethalom | Dunaharaszti, Szigethalom | **T _Water_ (°C)** | **pH** | **EC (µS/cm)** | **Dissolved Oxygen (mg/l)** | **Oxygen Saturation (%)** | **COD (mg/l)** | **Mineral N (mg/l)** | **Phosphate-P (µg/l)** |
| 15.12.2020 | 29.12.2020 | 12.01.2021 | 4.3 | 8.2 | 541.7 | 10.4 | 78.4 | 16.3 | 2.9 | 65 |
| 09.02.2021 | 23.02.2021 | 09.03.2021 | 5.3 | 8.1 | 509.2 | 11.2 | 85.7 | 16.8 | 3.3 | 65 |
| 20.04.2021 | 04.05.2021 | 18.05.2021 | 13.4 | 8.3 | 451.7 | 10.1 | 96.2 | 16.2 | 1.5 | 20 |
| 01.06.2021 | 15.06.2021 | 29.06.2021 | 20.4 | 8.1 | 366.7 | 8.5 | 90.7 | 15.5 | 1.3 | 45 |
| 13.07.2021 | 27.07.2021 | 10.08.2021 | 22.5 | 8 | 365.8 | 8.1 | 9.1 | 17.5 | 1.4 | 63.3 |
| 21.09.2021 | 05.10.2021 | 19.10.2021 | 14.9 | 8.1 | 493.2 | 8.6 | 77.6 | 18.4 | 1.9 | 50 |
| 02.11.2021 | 16.11.2021 | 30.11.2021 | 8.1 | 8.2 | 614 | 9.5 | 88.2 | 18.7 | 2.8 | 53.3 |
| 14.12.2021 | 28.12.2021 | 11.01.2022 | 3.1 | 8.1 | 533 | 9 | 71.3 | 18.6 | 2.7 | 68.3 |
| 25.01.2022 | 08.02.2022 | 22.02.2022 | 3.4 | 8.2 | 522 | 10.7 | 83.8 | 20.3 | 2.5 | 34 |
| 08.03.2022 | 22.03.2022 | 05.04.2022 | 6.9 | 8.6 | 484.5 | 10.3 | 79.7 | 17.8 | 2.1 | 20 |
| 19.04.2022 | 03.05.2022 | 17.05.2022 | 15.5 | 8.6 | 397.3 | 11.4 | 115 | 19.8 | 1.3 | 10.2 |
| 14.06.2022 | 28.06.2022 | 12.07.2022 | 21.9 | 8.1 | 360.5 | 8.8 | 100.8 | 18.5 | 1.1 | 63.3 |
| 26.07.2022 | 26.07.2022 | 23.08.2022 | 24.9 | 8 | 424.8 | 7.5 | 80.7 | 21.1 | 1.3 | 76.7 |
| 24.10.2023 | 14.11.2023 | 28.11.2023 | 10.3 | 8 | 495.3 | 9.3 | 93.8 | 19.8 | 2.2 | 71.7 |
